# On the identity of some weevil species described by Johann Christian Fabricius (1745–1808) in the Museum of Zoology of Copenhagen (Coleoptera, Cucujoidea, Curculionoidea, Tenebrionoidea)

**DOI:** 10.3897/zookeys.451.8462

**Published:** 2014-11-03

**Authors:** Miguel A. Alonso-Zarazaga

**Affiliations:** 1 Departamento de Biodiversidad y Biología Evolutiva, Museo Nacional de Ciencias Naturales (CSIC), José Gutiérrez Abascal, 2, E-28006 Madrid, Spain

**Keywords:** Weevils, Attelabidae, Brentidae, Curculionidae, Erotylidae, Mycteridae, Salpingidae, Tenebrionidae, *Bruchela*, *Chlorophanus*, *Holotrichapion*, *Temnocerus*, Johann Christian Fabricius, new species, new combinations, new synonymies, morphology, systematics

## Abstract

The types of thirty-two nominal weevil species described by Johann Christian Fabricius are reviewed and lecto- and paralectotypes are designated for twenty-two of them. A neotype is designated for *Curculio sticticus* Fabricius, 1777. *Protapion varipes* (Germar, 1817) is declared a *nomen protectum* over *Curculio flavipes* Fabricius, 1775. Based on a study of syntypes, *Rhinomacer curculioides* Fabricius, 1781 is confirmed as a member of *Mycterus* (Mycteridae), *Bruchus undatus* Fabricius, 1787 is tentatively transferred to Erotylidae, *Curculio fulvirostris* Fabricius, 1787 and *Anthribus roboris* Fabricius, 1798 are confirmed as members of *Salpingus* (Salpingidae), and *Brachycerus cristatus* Fabricius, 1798 is transferred to Tenebrionidae. Based on lectotype designation, *Curculio caninus* Fabricius, 1792 is confirmed as a synonym of *Sitona lineatus* (Linnaeus, 1758) and *Curculio innocuus* Fabricius, 1802 as a synonym of *Cneorhinus barcelonicus* (Herbst, 1797). *Bruchus rufipes* Fabricius, 1792 is not considered an available species name, but a later use of *Bruchus rufipes* Olivier, 1790. *Cossonus incisus* Pascoe, 1885 is reinstated as valid from synonymy under *Cossonus illigeri* Champion, 1909 and *Cossonus vulneratus* Illiger, 1805 from synonymy under *Cossonus canaliculatus* (Fabricius, 1792) (a primary homonym of *Curculio canaliculatus* Olivier, 1791). *Cossonus canaliculatus* Fabricius, 1802 is a secondary homonym of the former and is replaced with *Cossonus incisus*. *Salpingus fulvirostris* (Fabricius, 1787) is reinstated as valid from synonymy under *Salpingus planirostris* (Fabricius, 1787), a primary homonym of *Curculio planirostris* Piller & Mitterpacher, 1783. The following new combinations are proposed: *Brachysomus erinaceus* (Fabricius, 1802) (from *Curculio*), *Bronchus ferus* (Gyllenhal, 1840) (from *Hipporhinus*), *Bronchus glandifer* (Fabricius, 1792) (from *Curculio*), *Bronchus nivosus* (Sparrman, 1785) (from *Curculio*), *Bronchus sparrmani* (Gyllenhal, 1833) (from *Hipporhinus*), *Coelocephalapion atrirostre* (Fabricius, 1802) (from *Attelabus*), *Nerthops sticticus* (Fabricius, 1777) (from *Curculio*), *Piezotrachelus crotalariae* (Fabricius, 1802) (from *Attelabus*), and *Poropterus granulatus* (Fabricius, 1802) (from *Curculio*). The junior homonym *Brachycerus uva* Fabricius, 1792 (non Sparrman, 1785) is replaced by *Brachycerus fabricii* **nom. n.** The following new synonymies are established: *Brachycerus obesus* (Fabricius, 1775) = *Curculio scalaris* Fabricius, 1777, **syn. n.**, *Brachyderes lusitanicus* (Fabricius, 1781) = *Curculio moratus* Fabricius, 1798, **syn. n.**, Brachypera (Brachypera) crinita (Boheman, 1834) = *Curculio striatus* Fabricius, 1787, **syn. n.**, *Brachysomus erinaceus* (Fabricius, 1802) = *Brachysomus villosulus* (Germar, 1824), **syn. n.**, *Bronchus abruptecostatus* (Gyllenhal, 1833) = *Curculio spectrum* Fabricius, 1802, **syn. n.**, *Bronchus nivosus* (Sparrman, 1785) = *Curculio recurvus* Fabricius, 1802, **syn. n.**, *Camptorhinus tibialis* (Sparrman, 1785) = *Rhynchaenus alienatus* Fabricius, 1802, **syn. n.**, *Coelocephalapion atrirostre* (Fabricius, 1802) = *Coelocephalapion luteirostre* (Gerstäcker, 1854), **syn. n.**, *Cyrtoderes cristatus* (DeGeer, 1778) (Tenebrionidae) = *Brachycerus cristatus* Fabricius, 1798, **syn. n.**, *Desmidophorus hebes* (Fabricius, 1781) = *Curculio tuberculatus* Fabricius, 1792, **syn. n.**, *Donus salviae* (Schrank, 1789) = *Curculio denticornis* Fabricius, 1798, **syn. n.**, *Exomias holosericeus* (Fabricius, 1802) = *Exomias chevrolati* (Boheman, 1842), **syn. n.**, *Nerthops sticticus* (Fabricius, 1777) = *Nerthops guttatus* (Olivier, 1807), **syn. n.**, *Phyllobius oblongus* (Linnaeus, 1758) = *Curculio mali* Fabricius, 1782, **syn. n.**, and *Rhinocyllus conicus* (Froelich, 1792) = *Bruchus punctatus* Fabricius, 1798, **syn. n.** *Bronchus synthesys* **sp. n.** is described to represent the concept of *Hipporhinus spectrum* sensu Marshall, 1904, a misidentification.

## Introduction

Johann Christian Fabricius (1745–1808), also known as the “Prince of Entomology”, was the most prominent entomologist of his time. His authority was enormous and so highly influential that later authors used to cite him as the author of others’ species (e.g., Linnaeus’ species). This favoured the establishment of the so-called “principle of authority”, which endangered the universality required by the scientific naming of animals and which was fought by Hugh E. Strickland and others; eventually this reaction led to the creation of the International Commission on Zoological Nomenclature in 1895 and the adoption of an International Code of Zoological Nomenclature since 1905 (as the *Règles* in the beginning) (Melville 1995). Fabricius was a prolific describer for the standards of his time. He published 696 available species names and 302 new combinations (mostly with genera of his own). Some of these are still in their original combination and have never been reviewed.

In recent times, it has become clear that some kind of catalogue of the living beings on Earth should be prepared, not only for primary scientific use but also as a powerful tool for conservation management, pest control, etc. (Wilson 2003). Several initiatives are working towards this goal (e.g., Species 2000, GBIF, Encyclopedia of Life) even if the dispersal of energies and funds is regrettable. In the framework of the two first of these mentioned initiatives, Christopher H. C. Lyal (Natural History Museum, London) and I united efforts towards the creation of an electronic catalogue of all taxa of Coleoptera Curculionoidea (the Electronic Catalogue of Weevil names), now dubbed “WTaxa” and reachable at URL http://wtaxa.csic.es. This database is still being built and improved, containing now some 167000 names, including those of fossils, and deemed to contain almost all names published by 31^st^ December 2012.

The database was originally compiled from secondary sources and we are currently checking the original sources, a task not without its surprises as we found several cases of species absent from the currently cited original source, species available from the original source but never recorded later, and species that at a given moment in the story of entomology disappeared from the catalogues. The aim of the present paper is to clarify the identity of several of these “lost species” published by J.C. Fabricius by examination of their type specimens. These species are either not recorded in the Junk-Schenkling Coleopterorum Catalogus or are recorded with uncertainty about their identity (usually by the use of a question mark or a placement *incertae sedis*). This list is not complete, as several problems compound the location and identification of syntypes, and Fabrician type specimens in British collections have been studied only fragmentarily. In one case the discovery of a Fabrician type was communicated to an appropriate specialist and he proceeded to a proper identification (Prena 2005). In a few cases, Apionidae and Nanophyidae specimens were also checked to confirm their identity, as this was required for the first volume of Curculionoidea of the Catalogue of Palaearctic Coleoptera (Alonso-Zarazaga 2011b,c). The study of two of the species was advanced because of coincident interests (Caldara et al. 2012).

## Materials and methods

The holding of Fabricius’ specimens in Copenhagen is, for historical reasons, divided into two different collections, although these are now placed one after the other in the same drawer, following the page order of the Systema Eleutheratorum. The “Kiel collection” is on permanent loan, and the “Copenhagen collection” is basically the Sehestedt & Tønder Lund collection. Zimsen (1964) provided more information on this aspect.

In this paper, 32 species are studied, based primarily on specimens located at the Museum of Zoology of the University of Copenhagen and the results published to be incorporated to WTaxa. Lectotypifications are made when considered necessary to fix the concept of a nominal species. I have extensively used the information given by Zimsen (1964), although in some cases this was found to be inexact or incomplete. I have also included isolated comments in square brackets, when needed. Any author revising a genus containing Fabrician species should verify that the type specimens correspond to the current concept of these species. This may not hold in all cases, as it has happened in the genus *Bronchus* Germar, 1817.

The treatment of every species includes the original reference, the original statement of locality or other data related to type specimens, comments and a summary of the present status of the species. Label lines are separated by a slash (/). Dates provided are those recorded in WTaxa, which may differ from dates given in other works.

Specimens were photographed with an Olympus C7070WZ camera mounted on a photographic frame Kaiser RA1. Extended focus images were generated using the software CombineZP. The programs Adobe Illustrator CS5.0 and Adobe Photoshop CS5.0 were used for image postproduction and mounting.

## Results

### Potentially valid names

#### *Curculio flavipes* Fabricius, 1775

*Curculio flavipes* Fabricius, 1775: 133.

**Location.** Habitat frequens primo vere locis apricis calidioribus.

**Comments.** This nominal species has been currently placed in synonymy of *Apion varipes* Germar, 1817 or another member of genus *Protapion* Schilsky, 1908. Schilsky (1901, 1902) studied the single specimen and identified it as *Apion varipes*. However, he failed to give precedence to the Fabrician name, perhaps in the erroneous belief that *Curculio flavipes* DeGeer, 1775 (now in *Polydrusus*) has precedence. For the time being, it has been impossible to date DeGeer’s (1775) work and a date of 31^st^ December 1775 is to be assumed for it (Art. 21.3.2), whereas Fabricius’s (1775) tome has been dated as of 30^th^ April 1775 (Evenhuis 1997). However, the nominal species *Apion varipes* has been in constant use since its original publication (while the Fabrician one has been considered in synonymy of different species), and I therefore reverse their precedence under Arts. 23.9.1 and 23.9.2 of the Code.

Zimsen (1964) recorded the presence of one specimen in the “Kiel collection”. It carries an identification label as *Apion varipes* Germ. by Schilsky, who mentioned the characteristic apically curved front tibiae of the males of this species. The specimen is in a very poor state now, lacking the head, pronotum and elytra. Examination of the remaining parts supports the identification made by Schilsky, which is here considered correct because of the coloration of the mid and hind trochanters and tibiae, and the apex of penis not recurved in side view and slightly rounded-subtruncate in dorsal view. I designate this specimen as the lectotype and have added a white label with red margins and black writing: LECTOTYPUS / *Curculio flavipes* F. / Alonso-Zarazaga des. / 2014.

**Present status.** A *nomen oblitum* and a synonym of *Protapion varipes* (Germar, 1817), *nomen protectum*. The reversal of precedence is here made in accordance with Art. 23.9.2 by stating that, to my knowledge, *Curculio flavipes* Fabricius, 1775 (and its combinations) meet the requirements of Art. 23.9.1.1 and *Apion varipes* Germar, 1817 (and its combinations) meet those of Art. 23.9.1.2, quoting the following references: Abbazzi and Maggini 2009; Alonso-Zarazaga 1990; Alonso-Zarazaga 2011b; Braunert 2006; Cholokava 2008; Giovanleonardo and Osella 2001; Gønget 1997; Gurrea Sanz and Pérez Barroeta 1994; Hayat et al. 2002; Heijerman and Alders 2010; Khrolinskij 1965; Kocs 2010; Korotyaev et al. 1993; Mazur 2002; Merkl 2008; Morris 2003; Podlussány 1996; Scherf 1964; Schneider and Gruschwitz 2004; Sergeev 1977; Silfverberg 2004; Solodovnikova 1969; Ter-Minasian 1972; Wanat 2001; Zimsen 1964.

#### *Curculio sticticus* Fabricius, 1777

*Curculio sticticus* Fabricius, 1777: 227

**Location.** Habitat ad Cap. B. Spei.

**Comments.** This nominal species has been treated subsequently by Fabricius (1781: 191), Olivier (1791: 541), Fabricius (1792: 473, repeating the original description), Herbst (1795: 506), Fabricius (1802: 531) and Thunberg (1813: 382).

Zimsen (1964) stated the specimens are missing, which I can corroborate. However, in the ZMUC General Collection there is an old specimen labelled *Curculio sticticus*, which is not a type and belongs to *Nerthops guttatus* (Olivier, 1807), a most plausible identification, as it could have been compared to some syntype. I hereby designate this specimen as the neotype of this species and have added a handwritten red label: NEOTYPUS / Curculio / sticticus F. / Alonso-Z. 2008. The specimen is pinned through the fore half of the right elytron by a very fine needle, lacks the left fore leg from the middle of the femur and the whole left middle leg, but otherwise it is in a good state of preservation. It carries an old, whitish handwritten label: ff. [H?] stictica (underlined) / Cap. b. Spei. It is a small female, ca. 4 mm in length, with well separated dorsal patches, contrary to the original description, which mentions a pre-apical fascia, formed by the conjunction of several patches, as can be seen in some other specimens of this species. This neotypification is made here to fix the uncertainty about the use of *Curculio sticticus*, for taxonomic purposes (Art. 75.1).

**Present status.** This species is here accordingly transferred to the genus *Nerthops* Schoenherr, 1826, as *Nerthops sticticus* (Fabricius, 1777), comb. n. *Nerthops guttatus* (Olivier, 1807) is a new synonym of the former. The latter name has been used in the last 50 years only in a few catalogues, and does not qualify as a *nomen protectum* under Art. 23.9.2.

#### *Curculio scalaris* Fabricius, 1777

*Curculio scalaris* Fabricius, 1777: 228

**Location.** Habitat ad Cap. Bon. Spei. Dr. Schulz.

**Comments.** This nominal species was subsequently mentioned by Olivier (1790: 183) and by Herbst (1797: 87). Haaf (1957b: 552) treated *Brachycerus scalaris* (Fabricius) as a species unknown to him, while stating correctly that the types are in the Museum of Copenhagen. He cited Olivier’s treatment of *Brachycerus scalaris* as a misidentification in synonymy of *Brachycerus obesus* (Fabricius, 1775), following Schoenherr’s (1833b: 391) opinion.

I have found two syntypes in the “Kiel collection”, thus corroborating Zimsen’s (1964) statement. One carries a red label with TYPE printed on it, and a white label, partly printed and partly handwritten: Brachycerus / obesus F. / det. E. Haaf 1957. The other has a similar white label, only the determination date is 1960. I have compared them again with the single type specimen of *Curculio obesus* Fabricius, 1775 and found all three to be conspecific. The single type specimen of *Curculio obesus* also carries an identification label by Haaf dated 1960. It seems that Haaf checked these specimens after the publication of his monograph, but he never published any correction, as far as I know. I designate the first mentioned specimen as the lectotype and have added a white label with red margins and black writing: LECTOTYPUS / *Curculio scalaris* F. / Alonso-Zarazaga des. / 2014. The second specimen is a paralectotype and has a similar label.

**Present status.** This species is a new synonym of *Brachycerus obesus* (Fabricius, 1775). Olivier’s treatment is neither a different taxon nor a misidentification, just a transfer of the Fabrician species to the genus *Brachycerus* Olivier, 1789.

#### *Rhinomacer curculioides* Fabricius, 1781

*Rhinomacer curculioides* Fabricius, 1781: 199

**Location.** Habitat in Italia. Dr. Allioni.

**Comments.** This nominal species was later treated by Fabricius (1802: 428), who established the synonymy with *Mycterus griseus* [Clairville], 1798, and *Curculio rhinomacer* Paykull, 1792.

Zimsen (1964) reported four specimens in the “Kiel collection” and one in the “Copenhagen collection”. I have been able to study all five, which are to be considered syntypes. The “Copenhagen collection” specimen carries a label: Fabricius ded[it] / Mus. S. & T. L. / Rhinomacer / curculiono_ / des Fabr. All these specimens belong to the genus *Mycterus* [Clairville], 1798. Lectotypification should be made by a specialist in the group.

**Present status.** This is the species currently known as *Mycterus curculioides* (Fabricius, 1781) (Mycteridae).

#### *Curculio mali* Fabricius, 1782

*Curculio mali* Fabricius, 1782: 499

**Location.** Habitat Lipsiae. Dom. Prof. Leske.

**Comments.** This nominal species was subsequently treated by Fabricius (1792: 487), who indicated a synonymy with *Curculio padi* Bonsdorff, 1785 (a synonym of *Phyllobius pyri* (Linnaeus, 1758)) and by Herbst (1795: 261), Fabricius (1802: 542) and Olivier (1807: 415). Billberg (1820: 45) transferred it to the genus *Polydrusus*.

Zimsen (1964) reported the presence of two specimens in the “Kiel collection”, which I have studied. One belongs to Polydrusus (Metallites) marginatus Stephens, 1831, but it has a more modern green label with this correct name and does not match the original description, thus it is here deemed not to belong to the type series. The other belongs to *Phyllobius oblongus* (Linnaeus, 1758) (phenotype with dark elytra: ab. *floricola* Herbst, 1784) and matches the description, and I here designate it as the lectotype and have added a handwritten red label: LECTOTYPUS / Curculio / mali F./ Alonso-Z. 2008.

**Present status.** *Curculio mali* is a new synonym of *Phyllobius oblongus* (Linnaeus, 1758).

#### *Bruchus undatus* Fabricius, 1787

*Bruchus undatus* Fabricius, 1787a: 41

**Location.** Habitat in Africae floribus Dom. Vahl.

**Comments.** The description reads: “B. niger elytris fuscis: strigis undatis albis. Corpus medium, nigrum, immaculatum. Elytra laevia, fusca strigis tribus aut quatuor undatis fuscis.” Manuel (1797: 606) placed the species in the genus *Macrocephalus* Olivier, 1789 (Anthribidae), an invalid homonym, currently in synonymy of *Platystomos* Schneider, 1791.

Zimsen (1964) reported the presence of one specimen in the “Copenhagen collection” and of another in the “Kiel collection”. I have checked both. They each carry a label with the name *Tritoma undatum* and they are neither chrysomeloids nor curculionoids. The placement suggested by the labels seems to be a good approximation.

**Present status.** Apparently a member of Cucujoidea, probably in Erotylidae, that should be studied by a specialist. The species is not included in the recent volumes of the Catalogue of Palaearctic Coleoptera.

#### *Curculio flavescens* Fabricius, 1787

*Curculio flavescens* Fabricius, 1787a: 112

**Location.** Habitat in America meridionali Dom. Schreber [error, Wibmer and O’Brien 1986: 22]. A Western Palaearctic species.

**Comments.** This nominal species was later discussed by Olivier (1791: 528), Fabricius (1792: 454), Herbst (1795: 135) and Fabricius (1802: 512). Billberg (1820: 45) transferred it to *Brachyrhinus* and Schoenherr (1826: 54) to *Chlorophanus*, adopting, however, Herbst as author of the species. Schoenherr (in Ménétriés 1832: 214) described *Chlorophanus graminicola* and placed *Curculio flavescens* Herbst as a synonym of it, a fact reflected in the treatment given to the species by Günther and Zumpt (1933: 70). Thus the synonymy has been known for a long time.

Zimsen (1964) recorded one specimen in the “Kiel collection”, which is a male of the species usually known as *Chlorophanus graminicola*, pinned, in a good general condition, but lacking the fore right tarsus, the onychium of the mid right tarsus and that of the hind left tarsus. I here designate it as the lectotype and have placed a red handwritten label: LECTOTYPUS / Curculio / flavescens F./ Alonso-Z. 2008, on its pin.

**Present status.** A senior synonym of *Chlorophanus graminicola* Schoenherr, 1832. The nominal species *Chlorophanus graminicola* Gyllenhal, 1834 is a homonym and synonym of the former (synonymy by Schoenherr 1840b: 429). To my knowledge, Schoenherr’s name does not meet the requirements of Art. 23.9.2 to be declared a *nomen protectum*, so the correct name is *Chlorophanus flavescens* (Fabricius, 1787). This name has already been used as valid by Ren et al. (2013).

#### *Curculio fulvirostris* Fabricius, 1787

*Curculio fulvirostris* Fabricius, 1787b: 381

**Location.** Habitat in Scania Dom. de Paykull.

**Comments.** This nominal species was later treated by Fabricius (1792: 377) as a synonym of his *Curculio planirostris* Fabricius, 1787, when he transferred the latter to the genus *Anthribus*. Paykull (1800: 167) transferred *Curculio fulvirostris* to the genus *Anthribus*, and synonymized *Anthribus planirostris* under it.

Zimsen (1964) did not mention any type specimen and I have been unable to identify any either, probably because they are merged with the type material of *Curculio planirostris* Fabricius, 1787. This name is an invalid primary homonym of *Curculio planirostris* Piller & Mitterpacher, 1783 [a synonym of *Tropideres albirostris* (Schaller, 1783)].

**Present status.** A species belonging to Salpingidae. *Salpingus planirostris*, being an invalid primary homonym, has been incorrectly used as valid by Pollock and Löbl (2008). The correct name for this species is *Salpingus fulvirostris* (Fabricius, 1787).

#### *Bruchus rufipes* Fabricius, 1792

*Bruchus rufipes* Fabricius, 1792: 373

**Location.** Habitat Parisiis. Mus. Dom. Bosc.

**Comments.** The original description reads “*Bruchus rufipes* Oliv. Ins. tab. fig.”, so there is at least some doubt whether this is to be considered a different nominal species from *Bruchus rufipes* Olivier, 1790. This corroborates my suspicion that the figs of Olivier’s *Entomologie* (at least those for the weevils) were available with names before 1792 and used by Fabricius. The Fabrician “species” was synonymized by Germar (1819: 119) with *Anthribus sericeus* Fabricius, 1802 and placed in *Bruchela* by Dejean (1821: 78).

Zimsen (1964) did not treat this “species”, supporting my assessment that it is to be treated just as a later use of Olivier’s. Wolfrum (1929) also treated it as such.

**Present status.** A later use of *Bruchus rufipes* Olivier, 1790 (now in *Bruchela* Dejean, 1821), not a different nominal species.

#### *Brachycerus uva* Fabricius, 1792

*Brachycerus uva* Fabricius, 1792: 383

**Location.** Habitat ad Cap. Bon. Spei Dom. de Paykull.

**Comments.** This nominal species was later treated by Herbst (1797: 86), Fabricius (1802: 416), Thunberg (1813: 397) (who synonymized it with his *Brachycerus uva* Thunberg, 1799), and Billberg (1820: 39). Schoenherr (1833: 402) treated all the descriptions and previous uses of *Brachycerus uva* and *Curculio uva* Sparrman, 1785 as a single entity, with a description by Gyllenhal. However, Schoenherr (1840a: 673) later determined that his previous treatment of *Brachycerus uva* and its former synonyms involved different species and he named his own 1833 species as *Brachycerus racemus* Gyllenhal, 1840 and the Thunbergian species as *Brachycerus labrusca* Gyllenhal, 1840, keeping the name *Brachycerus uva* for the Fabrician species, which included the synonym *Curculio uva* Sparrman. However, Haaf (1957a: 119) did not consider Fabricius’ species a different nominal species from that of Sparrman, even if in the original description the latter is not mentioned as the source for either the name or the material, which was credited to Paykull.

Zimsen (1964) mentioned one specimen being in the “Kiel collection”. This specimen is a black, small (ca. 7.5 mm) *Brachycerus* that cannot be identified with any of the species included in Haaf’s (1957b) keys. It is close to the group of species related to *Brachycerus uva* (Sparrman, 1785) by the characters of the head and rostrum, however it differs from the species in this group by the absence of the characteristic patches of elongate yellow scales. In addition, it differs from *Brachycerus uva* (Sparrman) by the interstriae 3 and 5 being more prominently tuberculate than the others, which have almost obsolete tubercles, and the outer distal angle of fore and mid tibiae strongly prominent, knife-like. I have compared this specimen with representatives of the other species in the Copenhagen museum collection. I here designate this specimen as the lectotype and have placed a red, handwritten label: LECTOTYPUS / Brachycerus / uva F. / Alonso-Z. 2008, on its pin.

**Present status.** Being apparently a valid species, the name *Brachycerus uva* Fabricius, 1792 is a **new homonym** of that of Sparrman’s species, and is here replaced with *Brachycerus fabricii* Alonso-Zarazaga, nom. n.

#### *Curculio caninus* Fabricius, 1792

*Curculio caninus* Fabricius, 1792: 467

**Location.** Habitat in Germania Dom. Smidt.

**Comments.** This nominal species was subsequently treated by Herbst (1795: 496), Paykull (1800: 308) (who considered it to be a ‘variety’ of *Curculio lineatus* Linnaeus, 1758), Fabricius (1802: 524), Germar (1824: 416) (also as a ‘variety’ of *Sitona lineatus*) and Schoenherr (1826: 135) (who combined it with the genus *Sitona* as a valid species). However, Schoenherr (1834a: 110) placed it as a ‘variety’ of *Sitona lineatus* and Emden and Emden (1939) placed it in synonymy of *Sitona flavescens* (Marsham, 1802), with doubt.

According to Zimsen (1964) there are four specimens in the “Kiel collection”, which I have studied. Three of these belong to *Sitona lineatus* and one to *Sitona obsoletus* (Gmelin, 1790). Of the three *Sitona lineatus* specimens, one has a rather uniform yellowish brown, non-banded scaling and is here designated as the lectotype, as it closely matches the original description, except for the antennae not being black (an illusory character, as it often occurs in Fabricius’ descriptions), which in any case no other specimen has. The two other specimens are heavily banded and do not match the original description, and they are here deemed not to belong to the type series. The specimen of *Sitona obsoletus* has also a similar yellowish brown, non-banded scaly pattern and is considered to be a paralectotype. I have added handwritten red labels to each specimen as follows: lectotype, LECTOTYPUS / Curculio / caninus F./ Alonso-Z. 2008; paralectotype, similar, except for PARALECTOTYPUS. I have also added white identification labels.

**Present status.** The lectotype designation is made to avoid nomenclatural changes. Thus, this nominal species is confirmed as a synonym of *Sitona lineatus* (Linnaeus, 1758), as it has been recently treated by Velázquez de Castro (2013).

#### *Curculio glandifer* Fabricius, 1792

Figs 1–3

*Curculio glandifer* Fabricius, 1792: 483

**Location.** Habitat ad Cap. Bon. spei Mus. Dom. Lund.

**Comments.** This nominal species was subsequently treated by Herbst (1795: 511), Fabricius (1802: 537), Olivier (1807: 390), Thunberg (1813: 388) and Billberg (1820: 45), the last transferring it to his genus *Hipporhis*. Schoenherr (1833: 469) synonymized this species with *Hipporhinus spiculosus* Gyllenhal, 1833, with doubt. This doubtful placement was adopted by Schenkling and Marshall (1929).

Zimsen (1964) mentioned one specimen in the “Copenhagen collection”. I have studied it (ZMUC 00022544) and found it to be a male *Bronchus* pinned with a long, thin pin, in rather good state but lacking part of the right antenna and of the left front leg, and the whole left hind leg, and smeared with glue apparently used to stick the pronotum to the elytra. It carries a small, green, square label, a red label with TYPE printed and a handwritten label: Cap. bon. sp. / Mus. S. & T. L. / Glandifer / F. The specimen is similar to *Bronchus ferus* (Gyllenhal, 1840) (comb. n.) using Marshall’s (1904) key, but it differs from this species by the more elongate body outline, the oblong pronotum with fewer and larger tubercles, the elytral tubercles being devoid of scales on the apical half and well separated, not fused by the bases into a continuous crest, those on interstria 2 starting level with front margin of the white band or a little behind it, the fore tibiae curved at the apical third, the rostrum with the lateral keels low, rounded, and the epistome continuous with the dorsum of rostrum, not separated from it by a V-shaped sulcus. I have used for comparison specimens of *Bronchus ferus* identified by Marshall in the NHM collection. I designate this specimen as the lectotype and have added a white label with red margins and black writing: LECTOTYPUS / *Curculio glandifer* F. / Alonso-Zarazaga des. / 2014.

**Present status.** I consider that this is a valid species, *Bronchus glandifer* (Fabricius, 1792), comb. n., for the time being, until a modern revision may reveal its true affinities or identity.

**Figures 1–6.**
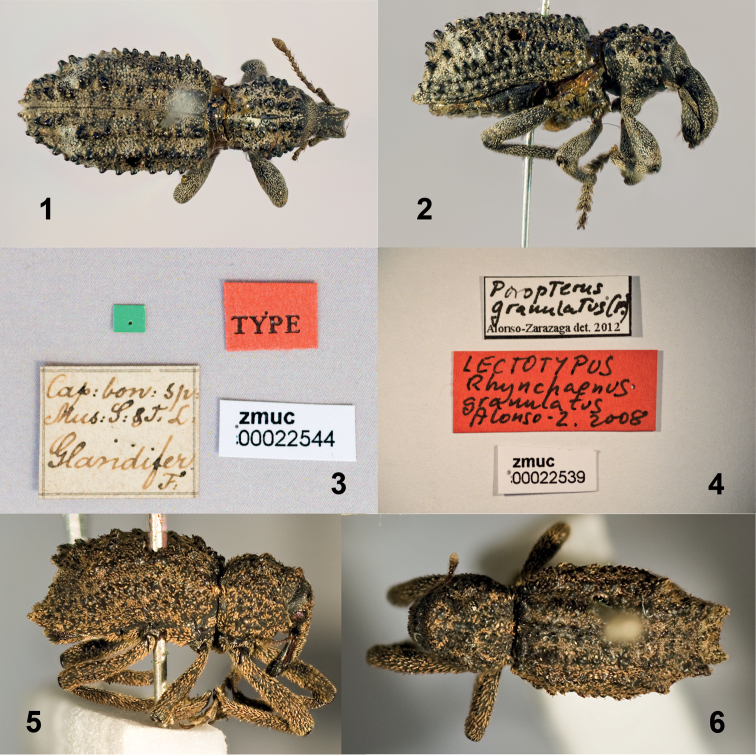
*Curculio glandifer* Fabricius lectotype **1** Dorsal view **2** Lateral view **3** Labels *Rhynchaenus granulatus* Fabricius lectotype **4** Labels **5** Lateral view **6** Dorsal view.

#### *Bruchus punctatus* Fabricius, 1798

*Bruchus punctatus* Fabricius, 1798: 158

**Location.** Habitat.

**Comments.** This species was subsequently treated only by Fabricius (1802: 397) and Schoenherr (1835: 153), who synonymized it with *Nerthops guttatus*, with doubt.

Zimsen (1964) reported the presence of two specimens in the “Kiel collection”, which I have been able to study. Both belong to the common species *Rhinocyllus conicus* (Froelich, 1792). I have selected the small one (apparently a male) as the lectotype and added the following handwritten red label to its pin: LECTOTYPUS / Bruchus / punctatus F./ Alonso-Z. 2008, and the larger one (apparently a female) as paralectotype, with a similar label except for the word PARALECTOTYPUS. They carry also white identification labels.

**Present status.** This is a new synonym of *Rhinocyllus conicus* (Froelich, 1792), and is removed from synonymy under *Nerthops guttatus* (Olivier, 1807) [erroneously as *guttula* in Klima (1935)]. Alonso-Zarazaga and Lyal (1999: 80) used *Bruchus punctatus* as the valid name for the type species of *Nerthops*, following the synonymy given in Klima (1935), but this is incorrect. See the treatment of *Curculio sticticus* above for more information.

#### *Anthribus roboris* Fabricius, 1798

*Anthribus roboris* Fabricius, 1798: 161

**Location.** Habitat in Kiliae Robore.

**Comments.** In the original description, Fabricius (1798: 161) pointed out that this was the same as *Attelabus ruficollis* Linnnaeus *sensu* Herbst, 1784, which is the case.

Zimsen (1964) reported a single specimen in the “Kiel collection”, which I have studied. It is a rather immature specimen of *Salpingus ruficollis* (Linnaeus, 1761). Lectotypification should be made by a specialist in the group.

**Present status.** This nominal species is a synonym of *Salpingus ruficollis* (Linnaeus, 1761) in Salpingidae. The current synonymy (cf. Pollock and Löbl 2008) is thus confirmed.

#### *Brachycerus cristatus* Fabricius, 1798

*Brachycerus cristatus* Fabricius, 1798: 161

**Location.** Habitat ad Cap. Bon. spei Mus. Dom. Lund.

**Comments.** This species was subsequently mentioned by Thunberg (1813: 399) and Schoenherr (1833b: 441), who transferred it to the genus *Sepidium* Fabricius, 1775 and listed as its synonyms *Tenebrio cristatus* DeGeer, 1778, *Brachycerus areolatus* Thunberg, 1799 and *Sepidium lacunosum* Thunberg, 1787.

Zimsen (1964) mentioned one specimen in the “Copenhagen collection”. I have checked it and, pending confirmation by a specialist in Tenebrionidae, consider it to be a syntype, and the nominal species to be an overlooked synonym of *Cyrtoderes cristatus* (DeGeer, 1778). This species is not recorded in the Coleopterorum Catalogus [Gebien (1910) did not include the name *Cyrtoderes* but did include as valid the genus *Phligra* Laporte, 1840, a junior synonym of *Cyrtoderes* Dejean, 1834 (Bousquet and Bouchard 2013); if present the species name *cristatus* would have been included under this genus].

**Present status.** A **new synonym** and secondary homonym of *Cyrtoderes cristatus* (DeGeer, 1778) (Tenebrionidae).

#### *Attelabus coeruleus* Fabricius, 1798

*Attelabus coeruleus* Fabricius, 1798: 163

**Location.** Habitat in Germania Dom. Daldorff.

**Comments.** This nominal species was later treated by Schoenherr (1833a: 232) as a synonym of *Rhynchites pauxillus* Germar, 1824. Dalla Torre and Voss (1937) placed it as a synonym of *Pselaphorhynchites tomentosus* (Gyllenhal, 1839). Legalov (2002) transferred the species to the genus *Temnocerus* Thunberg, 1815, without explanation.

Zimsen (1964) mentioned one specimen in the “Kiel collection”. I have studied what remains of it, identified as a *Temnocerus* and designated as lectotype by Legalov (2007: 124); it lacks the head and pronotum, left elytron, apical half of the right elytron and the hind left leg. The remains do not allow verification of the identification made by Legalov, he did not state whether he saw the whole specimen or just these remains.

**Present status.** Considered by Alonso-Zarazaga (2011a) as a species in the genus *Temnocerus*, *Temnocerus coeruleus* (Fabricius, 1798) (*fide* Legalov). However, the species is not recognisable from its lectotype and a neotype should be designated if considered necessary.

#### *Curculio moratus* Fabricius, 1798

*Curculio moratus* Fabricius, 1798: 171

**Location.** Habitat in Isle de France D. Billardiere

**Comments.** Later mentioned only by Fabricius (1802: 511).

Zimsen (1964) mentioned one specimen in the “Kiel collection”, which I have studied. It is a male of *Brachyderes lusitanicus* (Fabricius, 1781) in a very good state of preservation, pinned with a minuten pin and lacking the right antenna and the onychia of the right fore and hind tarsi. This specimen closely matches the original description, and I have selected it as the lectotype and added a red, handwritten label: LECTOTYPUS / Curculio / moratus F./ Alonso-Z. 2008 to its pin. Evidently the type locality given is a mistake, the species occurring in Spain, Portugal and western France.

**Present status.** *Curculio moratus* is a new synonym of *Brachyderes lusitanicus* (Fabricius, 1781).

#### *Curculio denticornis* Fabricius, 1798

*Curculio denticornis* Fabricius, 1798: 173

**Location.** Habitat ad Cap. Bon. spei Dom. Daldorff.

**Comments.** This nominal species has been later treated by Fabricius (1802: 539), who modified the provenance as: “Habitat in India orientali. Dom. Daldorff.”

Zimsen (1964) reported one specimen in the “Kiel collection”. This specimen (ZMUC 00022538) matches the original description and belongs in Hyperinae. It is pinned with a short, moderately thick, headless, somewhat bent pin to a piece of white plastic, which in turn is pinned with a long pin. The apical projection of the pedicel that gives its name to this species is an illusion. I have selected this specimen as the lectotype and placed the following red, handwritten label on its pin: LECTOTYPUS / Curculio / denticornis F./ Alonso-Z. 2008. It belongs to the genus *Donus* Jekel and is a normal specimen of *Donus salviae* (Schrank, 1789). The original locality and the subsequent correction have to be erroneous, as this is a Southern European species and not known to have been artificially dispersed beyond its original range.

**Present status.** A junior synonym of *Donus salviae* (Schrank, 1789), syn. n.

#### *Attelabus atrirostris* Fabricius, 1802

*Attelabus atrirostris* Fabricius, 1802: 424

**Location.** Habitat in America meridionali D. Smidt. Mus. D. de Sehestedt.

**Comments.** This nominal species was transferred by Schoenherr (1833a: 271) to the genus *Apion* Herbst, 1797 and has remained in it since then as an *incertae sedis* species.

Zimsen (1964) mentioned three specimens in the “Copenhagen collection” (seen by Kuschel) and one in the “Kiel collection”. I have been able to study all four, which are conspecific and belong in the genus *Coelocephalapion* Wagner, 1914. Of the three “Copenhagen collection” specimens, which are glued to the apex of small triangular card pieces, pinned with thin, long pins, the first (ZMUC 00523238) carries a label reading: Essequibo / Smidt / Mus: do. Sehestedt / Attelabus / atrirostris. This specimen is a female with a tubiform shiny rostrum, lacking the right part of the hind body. The second (ZMUC 00523239) is a male and lacks the abdomen and left elytron. The third (ZMUC 00523240) consists only of a pronotum, a mesosternum and some legs. The “Kiel collection” specimen (ZMUC 00523288), pinned with a thin, short pin going through a plastic piece, in turn pinned with a long pin, is in a better condition (even if the elytra and abdomen are parted), with a short brown rostrum, all legs and antennae intact, yellow, the latter inserted at the very base of the rostrum, big eyes, a very wide prothorax and the apex of the elytra slightly yellowish. This specimen cannot be considered a syntype, as it is not in the Sehestedt Collection. From the three syntypes in the Sehestedt Collection, I have selected the female carrying the Essequibo label as the lectotype and added a white label with red margins and black writing: LECTOTYPUS / *Attelabus atrirostris* F. / Alonso-Zarazaga des. 2014. The two other specimens are designated as paralectotypes and have accordingly ben fitted with similar labels. The type locality is here restricted to Essequibo, according to the label of the lectotype.

**Present status.** This is a valid species, *Coelocephalapion atrirostre* (Fabricius, 1802), comb. n., and *Coelocephalapion luteirostre* (Gerstäcker, 1854) is a new synonym of it (syn. n.). The latter name cannot be declared a *nomen protectum* for want of enough citations in the literature.

#### *Attelabus crotalariae* Fabricius, 1802

*Attelabus crotalariae* Fabricius, 1802: 424

**Location.** Habitat in Crotalariae leguminibus Americae. Mus. D. Lund.

**Comments.** Schoenherr (1833: 251), based on specimens apparently from the type series in his collection, transferred this species to the genus *Apion* Herbst, 1797.

Zimsen (1964) reported two specimens in the “Copenhagen collection” and one in the “Kiel collection”. I have studied the “Kiel collection” specimen and five specimens in the “Copenhagen collection”, all belonging to the same species of *Piezotrachelus* and matching Fabricius’ description. One of the males of the latter series carries a label: Essequibo / Isart[?] / Mus.: T. Lund / Attelabus / Crotalariae, and another label with an unpublished 1989 lectotype designation by M. Wanat. I have removed the latter label after consulting with Wanat and I added a white label with red margins and black writing reading: LECTOTYPUS / *Attelabus atrirostris* F. / Alonso-Zarazaga des. 2014, to this male, which I here designate as the lectotype. The other syntype specimens have been provided with similar paralectotype labels.

**Present status.** A valid species, *Piezotrachelus crotalariae* (Fabricius, 1802), comb. n., whose origin is doubtful, as this genus is not known to occur in America, while it is very common in Africa, and I suspect that a mislabelling has happened.

#### *Attelabus pisi* Fabricius, 1802

*Attelabus pisi* Fabricius, 1802: 425

**Location.** Habitat in Austria. Dom. de Meyerle.

**Comments.** This is a well-known species, transferred to the genus *Apius* by Billberg (1820: 40), to *Apion* Herbst, 1797 by Schoenherr (1833a: 304) and to *Holotrichapion* by Alonso-Zarazaga (1990: 127).

According to Zimsen (1964) there is only a name label in the “Kiel collection”. I have confirmed this situation, and consequently no type material is available in Fabricius’ collection. Johann Carl Megerle von Mühlfeld’s collection in Vienna should be checked, as it is possible that Fabricius returned this material to him.

**Present status.** A valid species, *Holotrichapion pisi* (Fabricius, 1802), very common and rather variable. For the moment, there is no need to designate a neotype.

#### *Rhynchaenus granulatus* Fabricius, 1802

Figs 4–6

*Rhynchaenus granulatus* Fabricius, 1802: 443

**Location.** Habitat in Amboina. D. Billardiere.

**Comment.** This species has not been mentioned in the taxonomic literature since description.

Zimsen (1964) reported the presence of a single specimen in the “Kiel collection”. This specimen (ZMUC 00022539) is a male Cryptorhynchini with most of the metanapleural sutures absent, the metanepisterna being visible only as a small triangular piece corresponding to the apex, the metaventrite between middle and hind coxae as long as the mesocoxal diameter, the crypt hind wall weakly prominent, almost perpendicular to the mesoventrite, the elytra tuberculate, with two prominent tubercles on top of the declivity and two others at the apex, the femora edentate and not ventrally sulcate, not reaching the elytral apex, the scutellum minuscule, with a few punctures bearing small scales, the antennal clubs troncoconical, velvety, with slightly oblique sutures, the funicles comprising seven desmomeres, the first two very long, the second slightly shorter than the first, the seventh subannexed to the club and rather similarly velvety, and the intermetacoxal distance much larger than the metacoxal width. I select this specimen as the lectotype and have added a red, handwritten label: LECTOTYPUS / Rhynchaenus / granulatus F./ Alonso-Z. 2008.

**Present status.** All these characters compel me to place this species in the genus *Poropterus* Schoenherr, 1844, as *Poropterus granulatus* (Fabricius, 1802), comb. n. This genus and its allies are in great need of revision. There is no other species of this genus recorded from Ambon or its adjacent islands, and the original locality could be incorrect, as the expedition commanded by Antoine Reymond Joseph Bruny d’Entrecasteaux (in which Jacques-Julien Houtou de La Billardière was enrolled as a naturalist) touched Australian lands at several places (La Billardière 1800) and a mislabelling could have happened.

#### *Rhynchaenus alienatus* Fabricius, 1802

*Rhynchaenus alienatus* Fabricius, 1802: 471

**Location.** Habitat in Sumatra. D. Daldorff.

**Comments.** This species has not been treated since the original description.

Zimsen (1964) mentioned the presence of one specimen in the “Kiel collection”. I have studied it (ZMUC 00022542), it is pinned with a short, rather thick pin in a piece of white plastic, which is in turn pinned with a longer pin. I designate this specimen as the lectotype and have added a red, handwritten label: LECTOTYPUS / Rhynchaenus / alienatus F. / Alonso-Z. 2008. It belongs to the genus *Camptorhinus* Schoenherr, 1825, showing a dark, wide sutural patch from the scutellum to middle of the elytra and it corresponds closely with specimens identified as *Camptorhinus porcatus* Fåhraeus in the NHM collections, a nominal species currently in synonymy of *Camptorhinus tibialis* (Sparrman). Even though the genus *Camptorhinus* needs a thorough revision, I propose a new synonymy here.

**Present status.** A synonym of *Camptorhinus tibialis* (Sparrmann, 1785), syn. n.

#### *Cossonus canaliculatus* Fabricius, 1802

*Cossonus canaliculatus* Fabricius, 1802: 496

**Location.** Habitat in Sumatra. D. Daldorff.

**Comments.** This species was mentioned later by Schoenherr (1826: 331; 1838: 1022).

Zimsen (1964) reported one specimen in the “Kiel collection” and two in the “Copenhagen collection”. I have seen all three, which are conspecific and belong to the genus *Cossonus* [Clairville], 1798 as usually understood. The “Kiel collection” specimen (ZMUC 00513702) is a male, pinned with a short, thick pin to a piece of plastic, which is pinned with a longer pin; the elytra are parted and it lacks the front left tibia and tarsus. The two “Copenhagen collection” specimens are males, glued to the apex of small triangular cards, but the elytra have big holes through which they were formerly pinned, in a similar way as the “Kiel collection” specimen, which cannot therefore be ruled out as a syntype. Specimen ZMUC 00513703 carries a small, green, square label and a written label: Sumatra / Daldorff / Mus. S. & T. L. / Cossonus / canalicula / tus Fabr. It is here designated as the lectotype, and I have added a white label with red margins and black writing to its pin: LECTOTYPUS / *Cossonus canaliculatus* / F. / Alonso-Zarazaga des. / 2014. The other specimen (ZMUC 00513704) has just the small green label. I have designated it and the “Kiel collection” specimen as paralectotypes, and added labels similar to that of the lectotype to their pins.

*Cossonus canaliculatus* (Fabricius, 1792) is an American species described as *Curculio canaliculatus* Fabricius, 1792 (*l.c.*: 471), which is a primary homonym of *Curculio canaliculatus* Olivier, 1791. It was correctly placed by Schoenherr (1838: 1030) as a synonym of *Cossonus vulneratus* Illiger, 1805, the reason for which was not clear to Champion (1909: 68), who apparently was unaware of the homonymy. The catalogues of O’Brien and Wibmer (1982: 222) and Wibmer and O’Brien (1986: 358) also used the incorrect name *Cossonus canaliculatus* (Fabricius, 1792). The name of the species treated here, *Cossonus canaliculatus* Fabricius, 1802, is thus a secondary homonym (because both species are now in the genus *Cossonus*) and cannot be used either. Csiki (1936: 166) used as its valid name *Cossonus illigeri* Champion, 1909 (a replacement name for *Cossonus canaliculatus* Fabricius, 1802), but the synonym *Cossonus incisus* Pascoe, 1885 has priority. These are to be considered provisional placements, as the genus *Cossonus* needs a comprehensive revision to establish its limits and contents. The synonymy is as follows:

*Cossonus incisus* Pascoe, 1885, stat. res.

= *Cossonus canaliculatus* Fabricius, 1802 (secondary homonym)

= *Cossonus illigeri* Champion, 1909 (replacement name)

*Cossonus vulneratus* Illiger, 1805, stat. res.

= *Curculio canaliculatus* Fabricius, 1792 (non Olivier, 1791)

**Present status.** A synonym of *Cossonus incisus* Pascoe, as shown above.

#### *Curculio innocuus* Fabricius, 1802

*Curculio innocuus* Fabricius, 1802: 512

**Location.** Habitat in Mogador. D. Schousboe. Mus. D. de Sehestedt.

**Comments.** This species was transferred by Schoenherr (1833b: 525) to his genus *Cneorhinus*, as var. β of *Cneorhinus barcelonicus* (Herbst, 1797).

Zimsen (1964) mentioned the presence of three specimens in the “Copenhagen collection”. I have examined them; each bears a small, green square and a red printed label with TYPE written on it. I have selected as lectotype the only female and added a red, handwritten label reading: LECTOTYPUS / Curculio / innocuus F. / Alonso-Z. 2008. This female carries a label reading: Mogador / Schousboe / Mus. Sehest. / Innocuus / Barcelonicus / Hbst. The two males are designated as paralectotypes and have been labelled accordingly. However, in the “Kiel collection” there is another specimen, which differs only in the claws being slightly more unequal. I do not consider it to belong to the type series, as this was composed only of specimens in Sehestedt’s collection.

**Present status.** A synonym of *Cneorhinus barcelonicus* (Herbst, 1797) as currently understood. The identity of this species nevertheless needs confirmation.

#### *Curculio holosericeus* Fabricius, 1802

*Curculio holosericeus* Fabricius, 1802: 526

**Location.** Habitat in Austria. D. Scheidler.

**Comments.** This nominal species was considered identical by Germar (1824: 405) with *Trachyphloeus ruficollis* (Fabricius, 1787) (a primary junior homonym) and was transferred by Schoenherr (1826: 192) to the genus *Omias* Germar, 1817. Schoenherr (1834b: 504) later synonymized it with *Omias rufipes* Boheman, 1834 (now in *Humeromima* Podlussány, 1998). However, Dalla Torre et al. (1937) placed it as a doubtful synonym of *Barypeithes indigens* (Boheman, 1834) (now in *Exomias* Bedel, 1883). It was treated as a valid species by Borovec (2013a: 84) replacing *Exomias indigens* (Boheman, 1834), but as a *nomen dubium* on the next page (*l.c.*: 85) and in the main text (*l.c.*: 382), showing that the poor original description does not allow a precise placement.

Zimsen (1964) mentioned a single specimen in the “Kiel collection”. I have studied it and identified it as a specimen of *Exomias chevrolati* (Boheman, 1842) in poor condition, matching the original description, with the abdomen and part of the metasternum missing, all the legs lost, except the mid left one showing the characteristic femoral tooth, the rostrum also with a characteristic strong convexity, the elytra only with moderately erect setae in one row per interstria, no appressed scales or pubescence. I here designate it as the lectotype and have added a red, handwritten label reading: LECTOTYPUS / Curculio / holosericeus F. / Alonso-Z. 2008 and a white identification label.

**Present status.** This is a valid species, *Exomias holosericeus* (Fabricius, 1802), with *Exomias chevrolati* (Boheman, 1842) as its new synonym. This species, originally described from Austria, has nothing to do with *Exomias indigens*, which is a South-Western European species.

#### *Curculio erinaceus* Fabricius, 1802

*Curculio erinaceus* Fabricius, 1802: 527

**Location.** Habitat in Austria. Dom. de Meyerle.

**Comments.** Billberg (1820: 44) was the first to propose a relationship of this species with the tribe Trachyphloeini, transferring the species to the genus *Trachyphloeus* Germar, 1817. Germar (1824: 412) suggested, with doubt, that it was a synonym of *Thylacites hirsutulus* (Fabricius, 1792), now in *Brachysomus* Schoenherr, 1823, whereas Schoenherr (1834b: 495) synonymized it with *Trachyphloeus horrens* Gyllenhal, 1834, also with doubt. Following the latter, Lona (1937) placed it in synonymy of *Cathormiocerus horrens* (Gyllenhal, 1834), with a question mark, and Borovec (2013b) recently placed it as a *nomen dubium* in the tribe Trachyphloeini.

Zimsen (1964) reported one specimen in the “Kiel collection” and two in the “Copenhagen collection”. The first, matching the original description, belongs to *Brachysomus villosulus* (Germar, 1824), having the scapes with the characteristic apical club of this species. I here designate it as the lectotype and have added a red, handwritten label reading: LECTOTYPUS / Curculio / erinaceus F. / Alonso-Z. 2008 and a white identification label. The two other specimens do not match the original description; both are males of *Exomias holosericeus* (cf. above), one carrying a label: Austria / Megerle / Mus. S. & T. L. / Erinaceus F. They are not considered to belong to the type series.

**Present status.** A valid species, *Brachysomus erinaceus* (Fabricius, 1802), comb. n., and a senior synonym of *Brachysomus villosulus* (Germar, 1824), syn. n. I have been unable to fulfil the requirements of Art. 23.9.1.2 to declare the latter name a *nomen protectum*.

#### *Curculio recurvus* Fabricius, 1802

Figs 7–9

*Curculio recurvus* Fabricius, 1802: 535.

**Location.** Habitat ad Cap. Bon. Spei. Mus. D. Lund.

**Comments.** This nominal species was transferred to the genus *Hipporhinus* Schoenherr, 1823 by Schoenherr (1840a: 753).

Zimsen (1964) recorded one specimen in the “Copenhagen collection”. I have studied it (ZMUC 00022545); it is pinned through the right elytron and carries a small, green, square label, a red printed label with TYPE and a whitish one reading: Cap: bon: sp: / Mus: S: & T. L. / Recurvus / F. It matches the original description, and I here designate it as the lectotype and have added the following red, handwritten label to ist pin: LECTOTYPUS / Curculio / recurvus F. / Alonso-Z. 2008. It is easily identifiable as *Bronchus nivosus* (Sparrman, 1785), comb. n., using Marshall’s (1904) key, and fits the description of the latter almost exactly. It is evidently not the same species treated as *Hipporhinus recurvus* by Marshall (1904), whose valid name is *Bronchus sparrmani* (Gyllenhal, 1833), comb. n. I have compared this specimen with specimens of both species identified by Marshall in the collection of the NHM (London).

**Present status.** A new synonym of *Bronchus nivosus* (Sparrman, 1785) in Marshall’s sense. The present problems with the identifications of the types of *Bronchus* make a modern revision of the genus necessary. Some of the characters used by Marshall in his keys, e.g., the “transverse basal furrow on the underside of rostrum” are highly variable among individuals of the same species, difficult to see and subject to different interpretations of the degree of depth, while other characters, e.g., the kind and disposition of vestiture on legs, are underused. The genitalia of both sexes have not been described.

**Figures 7–12.**
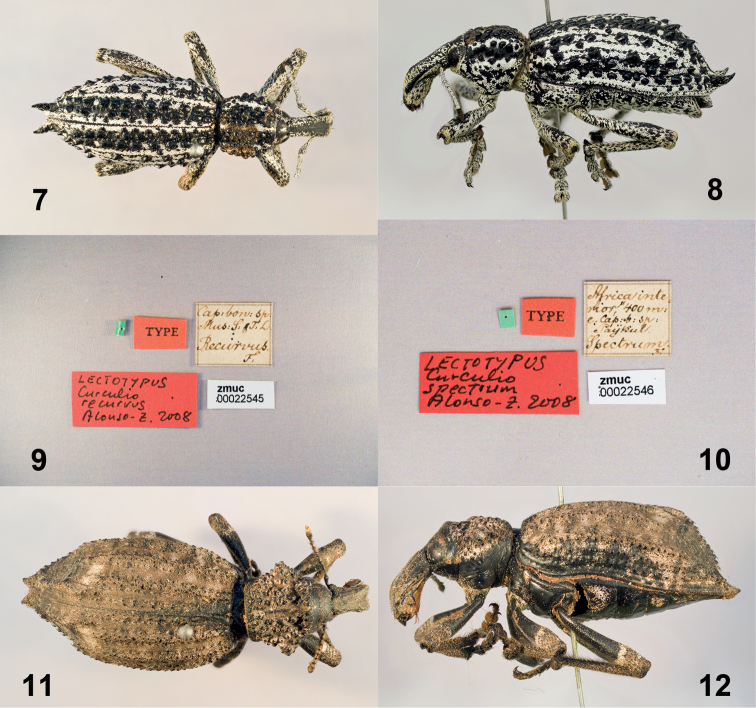
*Curculio recurvus* Fabricius lectotype **7** Dorsal view **8** Lateral view **9** Labels *Curculio spectrum* Fabricius lectotype **10** Labels **11** Dorsal view **12** Lateral view.

### Primary homonyms

#### *Curculio striatus* Fabricius, 1787

*Curculio striatus* Fabricius, 1787a: 117.

**Location.** Habitat in Africa Dom. Vahl [restricted by Fabricius (1792) to Barbaria].

**Comments.** This nominal species has been treated subsequently by Olivier (1791: 539), Fabricius (1792: 470), Herbst (1795: 501) and Fabricius (1802: 528). It was recently treated as a *nomen dubium* in Curculionoidea by Alonso-Zarazaga (2013a). This name is a primary homonym of *Curculio striatus* O.F. Mueller, 1776, of *Curculio striatus* Strøm, 1783 and of *Curculio striatus* Herbst, 1783 and is consequently invalid.

According to Zimsen (1964), there is one specimen in the “Copenhagen collection”, which I have studied. It is labelled: Vahl / Mus. S. & T. L. / “Striata”. It is a specimen of Brachypera (Brachypera) crinita (Boheman, 1834). I have added a handwritten red label reading: LECTOTYPUS / Curculio / striatus / Alonso-Z. 2008, and an identification label to its pin, and I here designate this specimen as the lectotype.

**Present status.** A new synonym of Brachypera (Brachypera) crinita (Boheman, 1834).

#### *Curculio planirostris* Fabricius, 1787

*Curculio planirostris* Fabricius, 1787a: 119.

**Location.** Habitat Kiliae Dom. Daldorff.

**Comments.** This species was later treated by Fabricius (1792: 377), Manuel (1797: 606) (who transferred it to the genus *Macrocephalus* Olivier, 1789), Paykull (1800: 167) (who placed *Curculio fulvirostris* Fabricius, 1787 as its synonym) and Fabricius (1802: 410). It was transferred to the genus *Rhinosimus* Latreille, 1802 by Latreille (1802) and to the genus *Salpingus* Illiger, 1802 by Illiger (1802). Its name is a primary homonym of *Curculio planirostris* Piller & Mitterpacher, 1783, a junior synonym of *Tropideres albirostris* (Schaller, 1783) (Anthribidae).

Zimsen (1964) reported three specimens in the “Kiel collection”, which I have studied and identified as *Salpingus planirostris* in the usual sense. I have not selected a lectotype from among the syntypes, preferring to leave this task for a specialist in the family Salpingidae.

**Present status.** A junior homonym, and consequently invalid, in use as *Salpingus planirostris* (Fabricius, 1787). Authors in Salpingidae seem to be unaware of this irregularity (e.g., Pollock and Löbl 2008). The correct name for this species is *Salpingus fulvirostris* (Fabricius, 1787) (see under this species above).

#### *Curculio tuberculatus* Fabricius, 1792

*Curculio tuberculatus* Fabricius, 1792: 480.

**Location.** Habitat in India orientali Dom. Prof. Abildgard.

**Comments.** This nominal species is a primary homonym of *Curculio tuberculatus* O.F. Müller, 1776 (a hitherto unidentified species) and of *Curculio tuberculatus* DeGeer, 1778 (a synonym of *Brachycerus obesus* Olivier, 1790). This species has been subsequently treated by Herbst (1795: 510) and Fabricius (1802: 535) without adding any clue about its correct placement.

Zimsen (1964) mentioned the presence of one specimen in the “Kiel collection”, which I have studied. It (ZMUC 00022543) is pinned with a thick, very short headless pin, which is pinned to a plastic piece, and this in turn is pinned with a long pin. The specimen is in relatively good state, despite suffering from a strong internal *Anthrenus* attack, but lacks the front left tarsus, the front right onychium and the right hind leg. The elytra are divaricate. I here designate this specimen as the lectotype and have added a red, handwritten label reading: LECTOTYPUS / Curculio / tuberculatus F. / Alonso-Z. 2008. This specimen belongs to the typical form of *Desmidophorus hebes* (Fabricius, 1781).

**Present status.** A new synonym of *Desmidophorus hebes* (Fabricius, 1781).

#### *Curculio spectrum* Fabricius, 1802

Figs 10–12

*Curculio spectrum* Fabricius, 1802: 537.

**Location.** Habitat ad Cap.Bon.Spei. Mus. D. Lund.

**Comments.** This nominal species is a primary homonym of *Curculio spectrum* Fabricius, 1781 (now in *Gyllenhalia* Aurivillius, 1886). It was transferred to *Hipporhinus* by Schoenherr (1833b: 462) and subsequently treated by Marshall (1904) in his revision of the genus, now a synonym of *Bronchus*.

Zimsen (1964) mentioned one specimen in the “Copenhagen collection”, which I have studied. It (ZMUC 00022546) is a female *Bronchus*, pinned through the right elytron, carrying a small, green, square label, a red printed label with TYPE, and a label reading: Africa inte / rior “ 400 m / e Cap: b: Sp: / Paÿkul / Spectrum. It shows *inter alia* two teeth on each side of the hind margin of the 4^th^ sternite and a laterally compressed declivity of the elytra, belonging therefore to *Bronchus abruptecostatus* (Gyllenhal, 1833). I here designate it as lectotype and have added a red, handwritten label reading: LECTOTYPUS / Curculio / spectrum F. / Alonso-Z. 2008. The unfortunate misidentification by Marshall leaves *Hipporhinus spectrum* sensu Marshall without a name, and I am describing it below as a new species.

**Present status.** A new synonym of *Bronchus abruptecostatus* (Gyllenhal, 1833).

### New taxon

#### Bronchus synthesys

Taxon classificationAnimaliaColeopteraCurculionidae

Alonso-Zarazaga sp. n.

http://zoobank.org/60096CFF-4B29-484D-BE2C-8769466414A9

##### Type material.

HOLOTYPE: 1 male, pinned, dissected, two legs absent, with the following labels: Marshall MS: Uitenhage / Cape Col. / Rev. J. ONeil; printed: G.A.K. Marshall / Coll. / B.M. 1950 – 255; Marshall MS: H. spectrum F. (Natural History Museum, London). This is one of the specimens coming from Marshall’s collection and mentioned in his 1904 revision. Despite its condition, it is considered to be the best specimen to meet the requirements for a holotype. A white label with red margins and black writing: HOLOTYPUS / *Bronchus synthesys* sp. n. / Alonso-Zarazaga des. / 2014 has been added to this specimen.

##### Description.

See that of *Hipporhinus spectrum* in Marshall (1904, pp. 54–55).

##### Etymology.

The specific epithet of this new species makes reference to the EC-funded project SYNTHESYS (http://www.synthesys.info) and is to be taken as a name in apposition. It pays homage to all the people who have made its operation possible.

##### Comments.

This is the species misidentified by Marshall (1904) as *Hipporhinus spectrum*. As it is a misidentified nominal species, its name is unavailable but cannot be replaced, as replacement names can only be proposed for available taxa (homonyms). Marshall’s species therefore is without a name, and I am describing it here.

## Discussion

Reviews of the type specimens in old collections usually have taxonomic implications. This article was not going to be an exception. It is linked to an international initiative (the World Information Network on Weevils), which aims at making available all kinds of information about Curculionoidea for use by researchers, applied entomologists (in Agronomy, Forestry and Food Storage) and decision-makers. One of its results is WTaxa. During the building and checking of this database, many ‘orphan species names’ were found, and there is a need to know the true identity of these nominal species.

The original list of species for this article was much longer. The problems faced during the research and the writing suggested that a reduction was necessary. Even so, six years of careful examination of all the available data resulted in thirty-two nominal species being treated here, twenty-two having lecto- and sometimes paralectotypes designated, one having a neotype designated to remove uncertainty about its identity and four illustrated for the first time. The nomenclatural changes proposed do not severely affect the taxonomy of species, since most of them have seldom been treated. Nine new combinations, a new replacement name and fifteen new synonymies are proposed, and one new species is described. Many other such ‘orphan species names’ await study, and some of the species will merit a full redescription and placement in new, still undescribed genera. On the other hand, it is not unusual to find that some species that have been treated by later authors, and even monographed, cf. *Bronchus* (as *Hipporrhinus*) by Marshall (1904), do not match their types, and this article may serve as a warning to monographers failing to check old types.

It is surprising that some of these species described long ago have apparently been only rarely collected since. Some (such as the *Bronchus* or *Brachycerus* species) have their habitats and ranges severely affected by human action and urban settlement, whereas others, bearing incorrect label data, may have been described from the real range of the species under a different name (e.g., *Piezotrachelus crotalariae*, *Poropterus granulatus*). In the end, this kind of study and its implications become archaeo-entomology, since we are studying a world that was but will never be again (cf. also Alonso-Zarazaga 2013b). It would be revealing to know how many of these species are already extinct. Let us hope not many of them.

## Supplementary Material

XML Treatment for Bronchus synthesys
